# Surveillance of Antibiotic Consumption Using the “Focus of Infection” Approach in 2 Hospitals in Ujjain, India

**DOI:** 10.1371/journal.pone.0038641

**Published:** 2012-06-08

**Authors:** Ashish Pathak, Kalpana Mahadik, Surya Prakesh Dhaneria, Ashish Sharma, Bo Eriksson, Cecilia Stålsby Lundborg

**Affiliations:** 1 Division of Global Health (IHCAR), Department of Public Health Sciences, Karolinska Institutet, Stockholm, Sweden; 2 Department of Pediatrics, R. D. Gardi Medical College, Ujjain, India; 3 Department of Obstetrics and Gynaecology, R. D. Gardi Medical College, Ujjain, India; 4 Department of Pharmacology, R. D. Gardi Medical College, Ujjain, India; 5 Department of Medicine, R. D. Gardi Medical College, Ujjain, India; 6 Nordic School of Public Health, Göteborg, Sweden; Johns Hopkins Bloomberg School of Public Health, United States of America

## Abstract

Antibiotic surveillance initiatives are limited in resource-constrained settings. In the present study, a quantitative comparison of antibiotic use rates for suspected infections in 2 hospitals in India was performed using the “focus of infection” approach to identify targets for quality improvement in antibiotic prescription patterns in hospitalized patients.

**Methods:**

This observational study was carried out in one teaching and one nonteaching hospital. All the patients with suspected bacterial etiology were included. Data on the prescribed antibiotics and the focus of infection were prospectively collected using a structured questionnaire. Each diagnosis was further reviewed and confirmed by an independent consultant. The prescribed antibiotics were coded according to the World Health Organization Anatomic Therapeutic Classification (ATC) index with the defined daily dose (DDD) methodology. Focus-specific DDDs were calculated per hundred patient days (DDD/HPD).

**Results:**

A total of 6026 patients were included from 72 participating physicians out of available 75 physicians. Overall antibiotic prescribing was higher by 5 percentage points in the teaching hospital (95%) than in the nonteaching hospital (90%). Quinolones (ciprofloxacin constituting 86% of DDD/HPD) were the highest prescribed class in the teaching hospital, and third-generation cephalosporins (with ceftriaxone and ceftriaxone/sulbactam constituting 40% and 28% of the DDD/HPD, respectively), in the nonteaching hospital. The targets identified for improvement were the following: longer than recommended duration of prophylaxis and lack of distinction between prophylaxis and therapy among surgical patients; irrational antibiotic prescribing in gastroenteritis; overuse of quinolones and lack of use of penicillin in pneumonia; overuse of quinolones and lack of use of doxycycline and macrolides in genital infections; and overreliance on antibiotics for treating skin and soft tissue infections.

**Conclusions:**

Providing a quantitative comparison of antibiotic use rates for suspected infections, using the “focus of infection” approach along with the ATC/DDD methodology, appears appropriate for identifying targets for quality improvement with regards to antibiotic prescribing.

## Introduction

Antibiotic resistance is a rapidly increasing public health problem [1]. Although most of the evidence is at an ecological level, it is widely accepted that the most potent driver for antibiotic resistance is antibiotic use [2]. The availability and use of the World health Organization (WHO) Anatomic Therapeutic Clinical classification and defined daily dose (ATC/DDD) methodology facilitate meaningful comparisons of antibiotic consumption across hospitals and also between countries [3]. An increase in the use of antibiotics in hospital settings has been documented worldwide, with a simultaneous increase in resistance and spread of resistant strains of many bacteria [4]–[7].

Disease surveillance projects have been initiated in several countries, and national antibiotic policies have been formulated. These projects, which used the ATC/DDD methodology, include Strama in Sweden [8], Danish Integrated Antimicrobial Resistance Monitoring and Research Programme (DANMAP) in Denmark [9], Dutch Working Party on Antibiotic Policy (Dutch acronym is SWAB) [10], Surveillance of Antibiotic Use and Resistance in Intensive Care (SARI) or Medical Antibiotic Use Surveillance and Evaluation (MABUSE) in Germany [11], European Surveillance of Antimicrobial Consumption (ESAC) in Europe [4], [12] and Intensive Care Antimicrobial Resistance Epidemiology (ICARE) in the United States [13]. However, there is a serious lack of similar initiatives in resource-constrained settings, where the burden of infections requiring effective antibiotics is higher [1], [7]. Because the cost of health care for resistant infections is high, injudicious use of antibiotics is a greater public health problem with respect to quality of patient care in resource-constrained settings. The National Centre for Disease Control, under the Director General of Health Services, Ministry of Health and Family Welfare, Government of India, published *The National Policy for Containment of Antimicrobial Resistance, India* in 2011 [14]. However, there are surprisingly few published reports describing the use of antibiotics in hospitals in India using the WHO ATC/DDD methodology. This lack of information hinders discussion of targeted interventions to reduce irrational antibiotic prescribing.

Accurate evaluation of antibiotic use can be achieved using patient-level surveillance [15]. However, even in the presence of accurate computerized prescription data, linking prescribing information to a given patient or diagnosis might not be possible. Extracting information from handwritten case records is time-consuming. In many resource-constrained settings, medical records are poorly maintained, leading to underestimation and misclassification of the underlying etiology associated with the prescription of antibiotics [16]. We have attempted to address these methodological challenges in the present study by using the “focus of infection” approach, along with the WHO ATC/DDD methodology, to study hospital antibiotic prescribing in a resource-constrained setting. The aim of this study was to provide a quantitative comparison of antibiotic use rates for suspected infections, using the “focus of infection” approach to identify targets for quality improvement with regard to antibiotic prescribing, taking 2 hospitals in Ujjain, India, as examples.

## Methods

### Study settings

The study sites were 2 hospitals. One is a 570-bed teaching hospital attached to the RD Gardi Medical College, which is a nonpaying facility located approximately 6 kilometers from the city of Ujjain. The consultants in this hospital have an institutional hospital-based practice and are not allowed to work in private practice. The other hospital is a 330-bed nonteaching hospital located in the city of Ujjain. In this hospital, all services are charged, but the hospital is run on a break-even basis. The consultants in the nonteaching hospital are allowed to work in private clinics outside of their official hours of work. Both hospitals cater predominantly to a rural population from the villages surrounding Ujjain city. In both hospitals, most admissions (91%) in medical and intensive care units are emergency admissions, whereas in the surgical units, both elective and emergency admissions are equally common. Treatment guidelines for infectious diseases have not been implemented in either hospital.

### Study participants

All the patients in whom the admitting consultant suspected an infectious etiology at the time of admission or during the hospital stay and for which antibiotic therapy was started were included in the study. The patients admitted for infectious etiologies requiring anti-infective agents other than antibiotics and those treated for tuberculosis were not included.

The participating departments or units in both hospitals were the departments of pediatrics, including the neonatal intensive care unit; general medicine, including the medicine intensive care unit; general surgery; obstetrics and gynecology; ear, nose, and throat; and orthopedics. A total 72 physicians out of available 75 from the above-mentioned departments participated in the study, a participation rate of 96%. Participation was voluntary.

### Sample size

The sample size calculation was based on a pilot study done in March and May 2007, which estimated that between 80 and 90% of all the admitted patients were prescribed antibiotics. Assuming a prevalence of 80% for antibiotic prescribing and requesting 80% power to detect a difference of 10 percentage points between hospitals for a given focus of infection in a statistical test for the comparison of 2 proportions with a 5% significance level, the required sample size was 108 in each of the focus of infection to be compared. Since, we had 15 foci of infection to be compared the minimum sample size was 15 Χ 108 Χ 2 = 3240 patients.

### Data collection form and procedure

The data collection form included patient details, age, sex, admission ward, dates of admission, discharge, prescribed antibiotic (s) (classified according to ATC), start and completion of antibiotic treatment course, dose per administration, number of doses per day, and route of administration. Any change in the antibiotic prescribed, its dose, or duration was noted. The final diagnosis was coded according to the targeted anatomical systems or subsystems identified as “focuses of infection” (Table 1) [4], [17]. Examples of typical infections in each group are also listed. The same diagnosis codes were used for surgical prophylaxis and for therapy. The duration of therapy for surgical prophylaxis was noted as single dose, single day, or more than 1 day.

**Table 1:** Details of anatomical system or sub-system identified as focuses of infection with examples of infectious diseases included in the study in two hospitals in Ujjain.

System/subsystem diagnosed as focus of infection	List of infections included in each focus of infection
CNS	Meningitis, meningo-encepalitis
Eye	Conjunctivitis, opthalmitis, retinitis etc
Ear, nose and throat, down to larynx	Tonsillitis, peritonsillitis, otitis, mastoiditis etc.
Bronchitis	
Pneumonia	Including pneumonia with septicemia,
CVS	Endocarditis, phlebitis, pericarditis
Upper gastrointestinal tract to terminal ileum	Peri operative prophylaxis, peritonitis, including H pylori
Lower gastrointestinal tract	Peri operative prophylaxis, peritonitis, intra-abdominal abscess of unknown origin, diverticulitis
Gastroenteritis	Gastro- intestinal tract, contagious diseases like Salmonella, Shigella, Vibrio cholerae etc.
Liver, biliary tract and pancreas	Hepatitis, cholecystitis etc.
Skin and soft tissue	Wound infections including post operative, erysipelas, deep infections, gangrene, myositis
Bone and joint	Osteomyelitis, arthritis etc
Renal	Pyelonephritis, febrile UTI, including uncomplicated urosepsis
Genital infections	Pelvic inflammatory disease, salpingitis, prostatitis, orchitis etc
Septicemia	Clinical evidence of sepsis
FUO/UFI	Focus of infection not identified
Unclear	Completely unclear indication
Health-care associated infection	Any infection resulting from any treatment or investigation associated with health care, regardless of whether the causing agent originates from the patient or the hospital environment

GIT gastrointestinal tract, FUO fever of unknown origin, UFI undifferentiated febrile illness, SSTI skin and soft tissue infections, ENT ear, nose and throat, CNS central nervous system, CVS cardiovascular system.

The questionnaire was attached to the inpatient files of all the admitted patients. The admitting consultant filled in the diagnosis codes. Each focus of infection was categorized into 1 of 3 indications for therapy or prophylaxis: (a) community-acquired infection; (b) hospital-acquired infection (for definition, see table 1); or (c) perioperative or medical prophylaxis.

The resident medical officers of the participating departments filled in the details of antibiotics prescribed by following up each patient from admission until discharge. A second independent consultant reviewed all the forms and also discussed and resolved any controversies regarding the focus of infection. The clinical suspicion of a focus of infection was confirmed by appropriate hematological, biochemical, and radiological investigations.

The structured questionnaire used in the present study was adapted from the Strama point prevalence surveys and ESAC [4], [17]. The final form was pilot tested twice in March and May 2007 on 100 patients each in both hospitals.

Each patient admission was counted only once, and patient transfers between units of a hospital were not counted as separate admissions. A patient's duration of stay was calculated by subtracting the date of admission from the date of discharge. The days of admission and discharge were counted together as 1 day. The same data collection form and procedure were used in both hospitals. Only 1 focus of infection, which was considered as the most relevant for therapy or prophylaxis, was included.

### Duration of data collection

Data collection was done from November 2007 to February 2009. The data were collected for 45 days in each hospital. A gap of 15 days was introduced between the 2 hospitals and every 4 months to allow time for appropriate data management. The study period covered 4 seasons, namely, 2 winters (1 in the beginning and 1 at the end, November to February), 1 summer (March to June), and the rainy season (July to October).

### Data management and statistical analysis

Each prescribed antibiotic was coded according to the WHO Collaborating Centre for Drug Statistics Methodology, ATC classification index with DDDs 2009 [3] as defined in the ATC fifth level in J01 (antibacterial for systemic use). Nitroimidazole derivatives (P01AB) and nitazoxanide (P01AX11) were also included. Focus-specific DDDs were calculated per hundred patient days (DDD/HPD). The DDD/HPD of a given focus of infection was compared to study the antibiotic prescribing pattern of the 2 hospitals.

The data were entered into the EpiData Entry (version 3.1) and then transferred to the Stata 10.0 for further analysis (Stata Corp., College Station, Texas, USA). A descriptive data analysis was conducted to understand the frequency of the patient-related variables (age groups, sex, services of the hospital, and season), foci of infection, and prescribed antibiotic and its class. The Pearson chi-square test was used to test for statistical significance (5%).

### Ethics statement

The ethics committee of RD Gardi Medical College approved the study (approval no. 41/2007). Informed written consent was obtained from all the patients. The research was conducted according to the principles expressed in the Declaration of Helsinki. The study did not interfere with the ongoing treatment of the patients.

## Results

The study included 6026 admitted patients, 2525 (42%) of whom were women and 3501 (68%) were men; 2352 patients (39%) were from the teaching hospital, and 3674 (61%) were from the nonteaching hospital. A total of 5531 patients (92%) were prescribed antibiotics for various foci of infection. Table 2 shows the details of the foci of infection recorded for the antibiotic treatment for the community-acquired infections, hospital-associated infections (HAI), and prophylaxis. Prophylaxis was given to 1846 patients (34% of the total), and in 86% (n = 1593), it was given for more than 1 day. Eighty-six percent of those on prophylaxis for more than 1 day belonged to surgical services, including the departments of general surgery; obstetrics and gynecology; ear, nose, and throat; and orthopedics. Gastroenteritis, pneumonia, bronchitis, central nervous system, and cardiovascular system infections and undifferentiated febrile illnesses were predominantly community-acquired. HAI was identified in 9% of all the infections. The most common HAI recorded was skin and soft tissue infection due to surgical site infection.

**Table 2:** Focuses of infection recorded for antibiotic treatment for community acquired and hospital-associated infections and for prophylaxis.

		No (%) of patients treated for		
Focus of infection	Total no of patients(n = 5531)	Community Acquired Infections(n = 3168) (57%)	Hospital Associated Infections(n = 517) (9%)	Prophylaxis(n = 1846) (34%)
Gastroenteritis	232	223 (96)	0	9 (4)
Upper and lower GIT	844	260 (31)	76 (9)	508 (60)
Liver, biliary tract and pancreas	133	78 (59)	18 (14)	37 (28)
Pneumonia	611	568 (93)	9 (3)	24 (4)
Bronchitis	191	174 (91)	6 (3)	11 (6)
Genital infections	665	173 (26)	51 (8)	441 (66)
FUO/UFI	559	445 (80)	30 (5)	84 (15)
Renal	495	218 (44)	79 (16)	198 (40)
SST	481	95 (20)	133 (28)	253 (53)
ENT and eye	291	192 (66)	8 (3)	91 (31)
CNS	262	232 (89)	22 (8)	8 (3)
Bone and joint infections	253	48 (19)	35 (14)	170 (67)
CVS	244	229 (94)	12 (5)	3 (1)
Sepsis	229	195 (85)	26 (11)	8 (3)
Unclear	41	38 (93)	2 (5)	1 (2)

GIT gastrointestinal tract, FUO fever of unknown origin, UFI undifferentiated febrile illness SSTI skin and soft tissue infections, ENT ear, nose and throat, CNS central nervous system, CVS cardiovascular system.

The patient's age group, sex, the hospital service, and the season in which the patient was admitted were significantly associated with antibiotic prescribing (Table 3). Most patients (59%) were prescribed 1 antibiotic. A combination of 2, 3, or 4 antibiotics was prescribed significantly more often in the teaching hospital. The mean length of stay in the teaching hospital was 7.8 days (95% confidence interval [CI] 7.3–8.3 days) and that in the nonteaching hospital was 4.2 days (95% CI 4.1–4.4 days).

**Table 3:** Patient characteristics and antibiotic prescribing in the two hospitals in Ujjain, India.

		Teaching hospital		Non-teaching	
Patient characteristics	Total PatientsN = 6026	Number of patientsn = 2352	% prescribedantibiotics (95)	Number of patientsn = 3674	% prescribed antibiotics (90)
**Age group**					
0–1 month	207	49	88	158	86
1 mo–5 years	539	151	88	388	88
6 years–12 years	294	123	86	171	88
13 years–45 years	3030	1206	95	1824	92
46 years–75 years	1795	765	97	1030	89
More than 75 years	161	58	97	103	90
**Sex**					
Females	2525	1026	95	1449	88
Males	3501	1326	95	2175	91
**Services**					
Paediatrics	686	192	81	492	87
Medical	2313	815	95	1498	90
Surgical	2628	1276	96	1352	90
NICU	192	30	97	162	90
ICU	207	39	97	168	92
**Season^(i)^**					
2^nd^ winter	1302	947	91	806	87
1^st^ winter	2189	580	96	772	91
Summer	782	587	97	1602	91
Rainy season	1755	238	99	544	90

i The study period covered four seasons, two winters, one in the beginning of the data collection and one at the end (November to February), summer (March to June) and the rainy season (July to October).

### Focus of infections

#### Gastroenteritis

In the teaching hospital, the prescribed antibiotics were ciprofloxacin (72 DDD/HPD), metronidazole (35 DDD/HPD), and cefotaxime (35 DDD/HPD). In the nonteaching hospital, ceftriaxone (40 DDD/HPD), ceftriaxone/sulbactam (4 DDD/HPD), ciprofloxacin (22 DDD/HPD), ofloxacin (20DDD/HPD), and metronidazole (30DDD/HPD) were prescribed (Table 4).

**Table 4:** Distribution of focus-specific DDDs prescribed per hundred patient days (DDD/HPD) for each antibiotic group in two hospitals in Ujjain, India.

Antibio tic groupsFocus of infection		Total patients in each diagnosis (DDDs are derived for hundred patients)	Co-trimoxazole J01EE01N1 = 7N2 = 111	Penicillin extended spectrum; Ampicillin, Amoxicillin and combinations with Cloxacillin J01CAN1 = 155N2 = 373	Penicillin with beta lactamase inhibitor Amoxi- cillin with clavulanic acid J01CRN1 = 183N2 = 82	Tetra- cyclines J01AN1 = 35N2 = 347	Cephalosporins; J01D		Lincos-amides* J01FF n = 32 and Macrolides J01FAN1 = 29N2 = 17	Quinolones J01MAN1 = 726N2 = 800	Metronidazole, Tinidazole Ornidazole Nitazoxanide (P01AX11) P01ABN1 = 449N2 = 506	Amino-glycosides J01GBN1 = 423N2 = 462	Other^$^N1 = 32N2 = 17	Total DDDs per hundred patients per focus of infection
							1^st^ Gen J01DB and 2^nd^ Gen J01DCN1 = 58N2 = 35	3^rd^ Gen J01DDN1 = 2443N2 = 765						
**Gastroenteritis**	**H1**	179	0	2	1	0	0	46	1	44	32	7	0	133
	**H2**	53	60	0	2	0	0	55	6	75	68	2	2	210
**Upper and lower GIT**	**H1**	391	0	9	7	5	5	93	0.5	61	83	29	2	294.5
	**H2**	453	140	22	7	54	4	48	24.4	83	122	43	0.2	407.6
**Liver, biliary tract and pancreas**	**H1**	93	0	32	2	0	4	88	0	48	56	33	4	267
	**H2**	40	0	38	13	28	10	25	0.5	45	48	3	0	210.5
**Pneumonia**	**H1**	379	0	7	14	1	3	57	5	32	4	4	3	130
	**H2**	232	276	41	11	69	0	22	1.4	42	11	29	0.4	226.8
**Bronchitis**	**H1**	42	0	7	7	2	0	36	0	7	0	0	0	59
	**H2**	149	86	21	8	112	3	25	1	40	11	14	0	235
**Genital infections**	**H1**	409	0	6	12	4	5	129	2	61	31	38	1	289
	**H2**	256	212	53	5	35	1	39	7	55	45	32	0	272
**FUO/UFI**	**H1**	457	14	4	5	6	2	75	2	29	8	7	1	139
	**H2**	102	141	11	5	7	0	60	5	25	3	7	3	126
**Renal**	**H1**	331	0	2	7	0	2	66	4.3*	93	19	59	1	253.3
	**H2**	164	298	8	4	0	1	55	23	93	17	50	2	253
**SST**	**H1**	227	236	6	9	4	7	92	17*	30	37	35	2	239
	**H2**	254	1361	28	12	18	8	44	18	40	41	58	0	267
**ENT and eye**	**H1**	79	0	8	8	0	8	65	3	8	9	14	4	127
	**H2**	212	253	24	0.5	6	1	42	1	94	11	36	1	216.5
**CNS**	**H1**	224	0	4	2	0	4	88	0.5	9	8	7	1	123.5
	**H2**	38	0	0	3	0	0	63	0	11	0	5	0	82
**Bone and joint infections**	**H1**	138	0	1	1	0	3	111	4	48	12	14	1	195
	**H2**	115	313	1	1	4	1	101	1	78	22	63	0	272
**CVS**	**H1**	165	0	13	6	1	1	66	1	8	1	1	0	98
	**H2**	79	0	14	10	3	0	67	0	18	5	20	0	137
**Sepsis**	**H1**	170	0	1	7	0	0.2	46	1	9	6	14	0.3	84.5
	**H2**	59	0	0	0	3	0	32	0	0.2	5	15	5	60.2
**Undefined**	**H1**	20	0	0	10	0	0	75	0	5	0	0	0	90
	**H2**	21	190	57	5	19	0	19	0	67	5	0	0	172

GIT gastrointestinal tract, FUO fever of unknown origin, UFI undifferentiated febrile illness, SSTI skin and soft tissue infections, ENT ear, nose and throat, CNS central nervous system, CVS cardiovascular system

Penicillin (J01CE) no prescriptions during the study period; Co-trimoxazole (J01EE01) the figures are in DUs (drug-units) and are not added to total DDDs

* Indications for which predominantly lincosamides were prescribed

H1 non-teaching hospital

H2 teaching hospital

N1 number of times each antibiotic group prescribed in non-teaching hospital

N2 number of times each antibiotic group prescribed in teaching hospital

#### Intra-abdominal (upper and lower gastrointestinal tracts)

The upper and lower gastrointestinal tracts were the most common sites of infection reported (n = 844) in the general surgery patients. Most of the patients (n = 508; 60%) received prophylactic antibiotic therapy, and 89% received it for more than 24 hours post surgery.

In the teaching hospital, nitroimidazoles were the most common antibiotics prescribed (122 DDD/HPD; DDD contribution: metronidazole 86%, tinidazole 12%, and ornidazole 2%), followed by quinolones (83 DDD/HPD-ciprofloxacin 88%, ofloxacin 10%, and norfloxacin 2%), tetracycline, and third-generation cephalosporins (Table 4).

In the nonteaching hospital, third-generation cephalosporins (93DDD/HPD-ceftriaxone 45%, ceftriaxone/sulbactam 42%, cefotaxime 8%, and ceftazidime 5%) were the commonest antibiotic class prescribed, followed by nitroimidazole (83DDD/HPD-metronidazole 68%, tinidazole 18%, ornidazole 9%, and nitazoxanide 5%; Table 4). Children were prescribed newly marketed nitazoxanide more often.

#### Respiratory (pneumonia and bronchitis)

In the teaching hospital, pneumonia was most commonly treated with tetracyclines (69 DDD/HPD-doxycycline 63% and tetracycline 37%), followed by quinolones (42 DDD/HPD-ciprofloxacin 94% and levofloxacin 5%), penicillins with extended spectrum (41 DDD/HPD-ampicillin with cloxacillin 76%, ampicillin 18%, amoxicillin 6%), and third-generation cephalosporins (22 DDD/HPD-cefotaxime 66% and ceftriaxone 24%); co-trimoxazole was also commonly prescribed. In the nonteaching hospital, third-generation cephalosporins (57 DDD/HPD-ceftriaxone with beta-lactamase inhibitor 56%, ceftriaxone 28%, and cefotaxime 12%), quinolones (32 DDD/HPD-ciprofloxacin 68% and levofloxacin 22%), and co-amoxiclav were prescribed.

**Figure 1:**
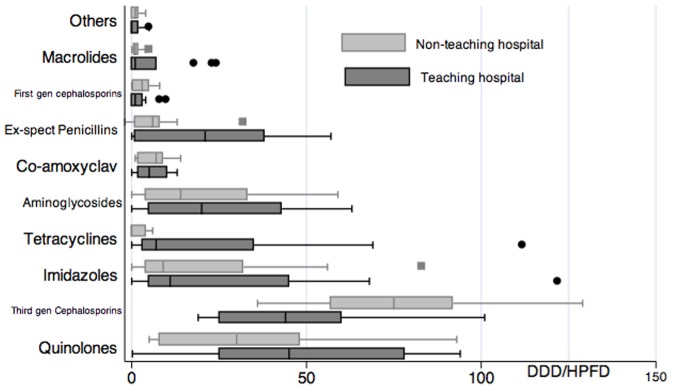
Wisker box plot comparing the antibiotic classes prescribed in Ujjain, India.

The prescribing pattern for bronchitis was similar to that for pneumonia in the respective hospitals (Table 4 and Figure 1).

#### Renal infections

In the treatment of renal infections, the antibiotic prescribing pattern was similar in the 2 hospitals. The quinolones, third-generation cephalosporins, and aminoglycosides were the most common classes prescribed. In the nonteaching hospital, however, a greater proportion of levofloxacin, ceftriaxone with a beta-lactam inhibitor, amikacin, and nitelmycin were prescribed (Table 4).

#### Genital infections

In the patients with genital infections (98% women), higher amounts of DDD/HPD were prescribed in the nonteaching hospital than in the teaching hospital (Table 4). In the teaching hospital, the most common antibiotics prescribed were quinolones (55 DDD/HPD-ciprofloxacin 64% and norfloxacin 33%), followed by penicillins with extended spectrum (53 DDD/HPD-ampicillin with cloxacillin 62%, ampicillin 33%, and amoxicillin 4%), nitroimidazoles (45 DDD/HPD-metronidazole 86%), and third-generation cephalosporins (39 DDD/HPD-cefotaxime 62% and ceftriaxone 32%). In the nonteaching hospital, third-generation cephalosporins (129DDD/HPD-cefotaxime 55%) and quinolones (61 DDD/HPD-ciprofloxacin 70%) were prescribed. Aminoglycosides and nitroimidazoles were prescribed often in combination with third-generation cephalosporins and quinolones.

#### Skin and soft tissue, bone, and joint infections

In both hospitals, skin and soft tissue infection (SSI) was most commonly treated with co-trimoxazole (1361 DU/HPD in the teaching hospital versus 236 DU/HPD in the nonteaching hospital). In the teaching hospital, aminoglycosides (58 DDD/HPD-gentamicin 65% and 32% amikacin), third-generation cephalosporins (44 DDD/HPD-cefotaxime 45% and ceftriaxone 34%), nitroimidazoles, and quinolones were prescribed (Table 4).

In the nonteaching hospital, the most common class prescribed was third-generation cephalosporins (92 DDD/HPD-ceftriaxone 36% or ceftazidime with beta-lactamase inhibitor 20%), followed by nitroimidazoles (37 DDD/HPD-metronidazole 69% and tinidazole 22%), aminoglycosides (35 DDD/HPD), and quinolones (30 DDD/HPD-ciprofloxacin 66% and levofloxacin 26%; Table 4).

For the treatment of bone and joint infections, the pattern of antibiotic prescribing was similar in the 2 hospitals (Table 4 and Figure 1), with third-generation cephalosporins, quinolones, and aminoglycosides being the top 3 classes prescribed (Table 4).

## Discussion

To the best of our knowledge, this is the first study to use the “focus of infection” approach, along with the WHO ATC/DDD methodology, for quantifying antibiotic prescribing in India. The DDDs were calculated per 100 patients per focus of infection per day (DDD/HPD). The results show that more DDD/HPD were prescribed in the teaching hospital for nearly all the foci of infections. These findings are similar to those of a large French study that demonstrated that higher antibiotic DDDs were dispensed in teaching hospitals [5]. The higher rate of antibiotic use in the teaching hospital in our study could be due to its status as a referral center for complicated cases, although disease severity was not monitored in our study. Newly marketed antibiotics like the combination of third-generation cephalosporins with beta-lactamases and Nitazoxanide, were prescribed more often in the non-teaching hospital. Since, in the non-teaching hospital services are provided for a cost and the teaching hospital is a free-of-cost facility, the payment status of the hospital is likely to influence the antibiotic prescribing. In a countrywide study among Irish general practitioners (GPs) it was found that GP's decision to provide a prescription for antibiotics might be influenced by whether or not the patient pays for their consultation {Murphy, 2011}. Combination antibiotic therapy was more common in the teaching hospital. However, the combination of third-generation cephalosporins with beta-lactamases, which is not a rational choice, was used more often in the nonteaching hospital. There is evidence that monotherapy is sufficient for all serious infections without shock [18]. Thus, developing guidelines for prudent antibiotic use for common infections is a priority identified through this study.

Approximately 9% of the infections were classified as HAI, which is a lower incidence than the expected rate of around 25% [19]. This might be due to the lack of specific surveillance for identifying HAIs and also to the lack of awareness of HAI. SSIs were the most commonly detected HAIs, and 28% of all the SSIs were surgical site infections. SSI was also a common reason for surgical prophylaxis. Establishing surveillance for HAI and specifically for SSI was also identified as a target for quality improvement in this study.

Approximately one third of all antibiotic prescribing was for prophylaxis, mainly in surgical services. Most (86%) of these patients received antibiotics for longer than 24 hours, which is a cause of concern that should be considered for possible intervention. Unnecessarily long surgical prophylaxis was also reported in the ESAC hospital point prevalence studies in 2006, 2008, and 2009 [12].

Variation in antibiotic prescribing according to age groups was observed in this study, with higher rates (by 4 to 9% unit points) of antibiotic prescribing in adults than in children. Such variation was documented previously in the same setting in an outpatient diagnosis prescribing study [20].

We analyzed the pattern of antibiotic prescribing in specific foci of infection. Over prescription of antibiotics for diarrhea is a major public health problem in India [21]. Acute diarrhea is self-limiting in most cases, and treatment should be restricted to rehydration, correction of electrolyte imbalance, and oral zinc for children [21]. Prescription of the nitroimidazole group of antibiotics, especially in children, cannot be explained rationally. The reasons behind such prescribing need to be researched, and appropriate, context-specific interventions need to be introduced.

Prophylactic use of antibiotics is common in patients undergoing surgical procedures [22]. A total of 508 (60%) of 844 patients received prophylactic antibiotics for prevention of infection localized to the upper and lower gastrointestinal tract. The most common antibiotic regimens were ciprofloxacin with metronidazole and third-generation cephalosporins with metronidazole in the teaching and nonteaching hospitals, respectively. Given the polymicrobial nature of intra-abdominal infections arising from resident enteric flora, the above-mentioned combination therapy might be justified but only as a treatment option for source control [22]. We observed no difference in the antibiotics chosen for treatment and prophylaxis. In addition, as already discussed, most prophylaxis continued beyond 24 hours after surgery. The lack of distinction between the use of antibiotic therapy for prophylaxis and its use for treatment of an established infection is identified as an important quality improvement target.

Doxycycline and tetracycline dominated the prescription pattern for pneumonia in the teaching hospital, and these are appropriate for community-acquired pneumonia (CAP) of all severities according to the British Thoracic Society guidelines [23]. The use of ciprofloxacin, which was the second most common antibiotic prescribed for CAP, is not appropriate especially in view of its poor activity against *Streptococcus pneumoniae* and *Klebsiella pneumoniae*, the 2 organisms most frequently associated with adult pneumonia in the Asia-Pacific region [24]. Non-use of penicillin is a cause of concern, as recent evidence shows that fewer than 5% of all the nonmeningeal isolates of *S. pneumoniae* from the Asia-Pacific are penicillin resistant (according to an MIC of 8 µg/mL) [24]. Convincing physicians to increase use of penicillin is identified as a key quality improvement issue.

The Infectious Disease Society of America recommends the use of 1 of the following 3 initial therapies for acute pyelonephritis in adults: (a) fluroquinolone, (b) aminoglycoside with or without ampicillin, or (c) extended spectrum cephalosporin with or without aminoglycoside [25]. The antibiotic prescribing pattern in renal infections appeared to be appropriate in both hospitals.

There is a significant unmet demand for the treatment of genital infections among women in rural India due to their high disease burden [26]. In addition, the skewed urban distribution of health-care workers contributes to this unmet demand [27]. The optimization of therapy for hospitalized genital infections (especially pelvic inflammatory disease) must take into account the polymicrobial etiology of the disease, the severity of the disease, and patient compliance with antibiotic use. The optimal treatment for genital infections should include an antibiotic with activity against *Neisseria gonorrhoeae*, *Chlamydia trachomatis*, and *Mycoplasma genitalium.* Because no single agent covers all of these organisms, combination therapy is recommended [28]. In the teaching hospital, ciprofloxacin or norfloxacin were prescribed, often in combination with metronidazole. However, ciprofloxacin is less effective against bacterial vaginosis-associated microorganisms; therefore, possible relapse of the infection is of concern. In addition, increasing quinolone resistance among *N. gonorrhoeae* isolates has been documented in the Asia-Pacific region [7]. Third-generation cephalosporins are a good addition to the regimen [28], which was used more often in the nonteaching hospital. Overuse of aminoglycosides and lack of use of doxycycline and macrolides were identified as quality improvement targets in antibiotic prescribing for genital infections.

Among hospital patients with SSI, antibiotic therapy should be initiated only if the patient fails to respond to incision and drainage or shows abscess with severe and extensive disease, rapidly progressive cellulitis, signs and symptoms of systemic disease, associated comorbidities, immunosuppression, or an abscess in an area difficult to drain (face, neck, or hand) [29]. Most of the patients treated with antibiotics in the 2 hospitals did not fulfill these criteria. The most common choice of antibiotic was co-trimoxazole, which appears appropriate. However, the relatively common use of quinolones and third-generation cephalosporins is not justified [29].

The main strength of the present study is that unlike most surveillance [4], [5], [6], [11], [12], which collects and presents dispensing data at aggregate levels, we collected information on individual patients and on antibiotics actually administered to the patients. Therefore, our data are robust and relevant for interventions and for monitoring trends. The validity of our data is strong because an independent expert confirmed the foci of infection. However, there are a few limitations. The design of the study is resource intensive, requiring dedicated personnel. We did not evaluate the severity of illness, which would have further intensified the workload. The study did not include positive bacterial cultures or other laboratory measures to confirm the focus of infection, but would like to do so in our future studies. Monitoring of sensitivity patterns itself can change prescription patterns. The results are not strictly comparable within a season, as data were not collected simultaneously in the 2 hospitals. In the present study we have focused on choice of antibiotics as the main outcome but other aspects of rational antibiotic prescribing like duration of treatment and appropriateness of choice of formulations (for example oral therapy versus intravenous and use of syrup or tablets) is not discussed in the study. The expectancy effect i.e. the consultants under observation changing the rate of antibiotic prescribing, is a potential bias. However, the prescribing was observed for a longer duration in this study, thus minimizing the expectancy effect.

### Conclusions

Using a data collection procedure that can produce good focus of infection-specific information on ATC/DDD, we were able to identify targets for quality improvement in antibiotic prescribing. The targets identified are higher antibiotic prescribing in a teaching hospital compared with a nonteaching hospital, longer than recommended duration of prophylaxis and lack of distinction between prophylaxis and therapy among surgical patients, irrational antibiotic prescribing in gastroenteritis, overuse of quinolones and lack of use of penicillin in pneumonia, overuse of quinolones and lack of use of doxycycline and macrolides in genital infections, and overreliance on antibiotics in treating skin and soft tissue infections. The study provides much needed Indian data for policy makers to design strategies for promoting prudent antibiotic use and formulating national, regional, and local therapeutic guidelines.
